# Hormesis and Female Sex Hormones

**DOI:** 10.3390/ph4050726

**Published:** 2011-05-16

**Authors:** Jakob O. Strom, Annette Theodorsson, Elvar Theodorsson

**Affiliations:** 1 Institution of Clinical and Experimental Medicine/Department of Clinical Chemistry, Linkoping University, Linkoping, Sweden; 2 Institution of Clinical and Experimental Medicine/Department of Neurosurgery, Linkoping University, Linkoping, Sweden

**Keywords:** hormesis, non-monotonic, biphasic, β-curve, estrogens, 17β-estradiol, progesterone, progestagens, hormone therapy, menopause

## Abstract

Hormone replacement after menopause has in recent years been the subject of intense scientific debate and public interest and has sparked intense research efforts into the biological effects of estrogens and progestagens. However, there are reasons to believe that the doses used and plasma concentrations produced in a large number of studies casts doubt on important aspects of their validity. The concept of hormesis states that a substance can have diametrically different effects depending on the concentration. Even though estrogens and progestagens have proven prone to this kind of dose-response relation in a multitude of studies, the phenomenon remains clearly underappreciated as exemplified by the fact that it is common practice to only use one hormone dose in animal experiments. If care is not taken to adjust the concentrations of estrogens and progestagens to relevant biological conditions, the significance of the results may be questionable. Our aim is to review examples of female sexual steroids demonstrating bidirectional dose-response relations and to discuss this in the perspective of hormesis. Some examples are highlighted in detail, including the effects on cerebral ischemia, inflammation, cardiovascular diseases and anxiety. Hopefully, better understanding of the hormesis phenomenon may result in improved future designs of studies of female sexual steroids.

## 1. Introduction

The concept of hormesis reflects the pharmacological phenomenon of a substance producing diametrically different effects depending on the dose, thus negating the notion that dose-response curves are generally unidirectional [1]. Although debated due to initially limited experimental evidence, the concept has been successively established as a relevant model for explaining the biological effects of certain substances [2]. Another controversial subject during recent years is the menopausal hormone therapy debate. The effects of estrogens on stroke have been especially conflicting, since large epidemiological studies [3-5] and numerous animal studies [6-10] have found hormone therapy to be neuroprotective, while, on the contrary, the randomized controlled trial Women's Health Initiative (WHI) [11] reported increased stroke risk and some animal studies [12-15] have demonstrated increased ischemic lesions. Recent evidence indicates that estrogens' effects in rat stroke models may obey hormetic principles, so that physiological concentrations are protective while higher, prolonged concentrations are detrimental [16,17].

With the current widespread consumption of female sex hormones as contraceptive pills and menopausal hormone therapy, it is crucial to acknowledge the potentially hormetic effect patterns when designing animal studies, assessing study data and when planning clinical trials. This may be done by using a wide range of doses and by, at several relevant time-points, measuring the resulting *in vivo* serum concentrations of the hormone. Although all this may seem obvious, these measures to ensure study quality are unfortunately very commonly neglected. For illustrative purposes, we performed a literature review, covering the latest 100 articles describing administration of 17β-estradiol to rats or mice, retrieved when searching Medline (on the 25th of March 2011) with the terms “Estradiol and (rat or mouse)”. Of these 100 articles, published in 2010 and 2011, 86 described administration of only one 17β-estradiol dose, and only seven studies adopted more than two doses. It is interesting to note that in the study using the highest number of doses, a clear bidirectional dose-response relation was seen [18]. Serum 17β-estradiol measurements were only performed in 27 of the studies, of which 25 only investigated one single time-point, which thus conveys little information about the serum concentrations before and after the exact moment the sample was taken (Table 1).

The aim of this review is to highlight the importance of taking hormesis into account in all studies investigating the biological effects of female sexual hormones. The fundamentals of hormesis are first described and discussed, and the definition adopted in this review is outlined (Section 2). Subsequently examples of female sex steroids demonstrating hormetic dose-response relations, including the abovementioned biphasic actions of estrogens in stroke, are presented (Section 3), followed by concluding remarks regarding the implications for menopausal hormone therapy research (Section 4).

## 2. The Concept of Hormesis

The first record of the term “hormesis” in scientific publications is found in the 1943 article by Southam and Ehrlich “Effects of extract of western red-cedar heartwood on certain wood-decaying fungi in culture”. The authors investigated the effects of a wide concentration range of an anti-fungal agent, finding that despite high concentration decreased the fungus growth, doses below the growth-inhibitory threshold actually stimulated it [19]. Thus, this original adoption of the term described the phenomenon that merely depending on the dose, one substance could have diametrically different effects in a biological system. However, although Southam and Ehrlich were the first to use the term “hormesis” in scientific publications, the phenomenon had been acknowledged much earlier. Actually, already the ancient Greeks' proverb “meden agan” (*nothing in excess*), the Latin analogue “in medio stat virtus” (v*irtue stands in the middle*), as well as Paracelsus well-known quote “Alle Dinge sind Gift und nichts ist ohne Gift, allein die Dosis macht es, dass ein Ding kein Gift ist” (*all things are poison and nothing is without poison; only the dose makes a thing not a poison*) reflects aspects of hormesis. The scientist most often attributed as the first to scientifically identify the hormetic phenomena, though without using the word “hormesis”, was Schultz, who in a series of studies as early as in the 1880's demonstrated e.g. that formic acid promoted fermentation in low doses while inhibiting it in higher doses [20].

Before and in parallel with the adoption of the term “hormesis” in the 1940's, numerous terms for similar phenomena were suggested, including “biphasic”, “bidirectional”, “non-monotonic”, “J-shaped”, “U-shaped” and “inverted U-shaped dose-response curves”, “β-curve”, “Arndt-Schultz' law” and “Huebbe's law”. The rich flora of terms has probably contributed to confusion and difficulty in properly investigating the phenomenon, thus the fundamental importance of clearly defining a term, such as “hormesis”, to precisely account for bidirectional dose-response relations of this sort cannot be overestimated.

As mentioned before, a lively debate concerning the definition and significance of hormesis has taken place in the scientific community in recent years [1,2,21,22]. One of the most influential scientists in the field is Calabrese, who has not only performed extensive literature analyses to assess the phenomenon's frequency and nature [23,24], but also in a series of reviews has revised the hormesis definition [2,25-28]. An important contribution by Calabrese in developing a scientifically sound definition of hormesis was the realization that the low-dose effect of hormesis should not necessarily be beneficial, since “beneficial” is an utterly complex and context-dependent expression [2].

A related question is if the mechanism(s) should be included in the definition of hormesis. In an attempt to more strictly define hormesis by attributing it to one common mechanism, it has been suggested that hormesis should be viewed as an adaptive action taken by the cell to minimize the damage from a toxic insult. This adaptation would in turn be direct or resulting from overcompensation by the toxic damage, in the latter case with a mandatory time delay [2,25,29]. However, it seems unnecessarily narrow to define one type of mechanisms for all types of hormetic dose-responses, as pointed out by Kendig *et al.* [1]. Further, such a definition is unintuitive, probably unrelated to many of the instances in which the term has been used, and the classification of a dose-response relation becomes exceedingly complicated if an adaptive nature of the response needs to be proven in every single case. Adaptation to toxic insults can definitely be the one possible mechanism for certain hormetic responses, but the concept of hormesis should not be limited to this. Instead, Kendig *et al.* suggested that the definition of hormesis should solely be related to the bidirectional dose-response curve, and unrelated to its mechanism: “Hormesis is a dose-response relationship for a single endpoint that is characterized by reversal of response between low and high doses of chemicals, biological molecules, physical stressors, or any other initiators of a response” [1]. In line with this definition, Conolly and Lutz illustratively demonstrated how different multi-mechanistic systems, including adaptations to damage, can render hormetic dose-response curves for certain endpoints. They thus highlighted that hormesis is most likely to occur in mechanistically complex systems, where a multitude of mechanisms with different potency and efficiency taken together can create a bidirectional pattern [30]. The advantage of this definition is that it is intuitive, readily enables identification of hormesis and is far less speculative than the above mechanism-coupled definition.

**Figure 1:**
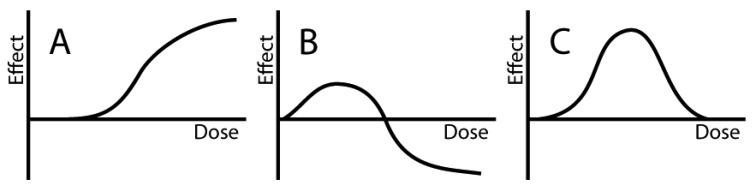
The classical linear/threshold dose-response relation is due to its monotonic behavior **(A)** clearly distinct from the non-monotonic hormetic pattern. **(B)** However, not all non-monotonic dose-response relations are hormetic, exemplified by the unidirectional (producing effects on only one side of the baseline), non-monotonic relation presented to the right **(C)** which is not an example of hormesis.

It should be also emphasized that not all non-monotonic dose-response curves are included in the concept of hormesis, but that effects in both directions compared to the control group need to be demonstrated (Figure 1).

The debate concerning the nature of hormesis has largely been conducted within the realm of toxicological sciences, which has influenced the suggestions of how the term should be used. For example, the hormetic effect has most often been described as the sub-threshold stimulatory effect of a dose-toxicity curve, rather than e.g. the reversal of a drug's desired effect in doses above the therapeutic window [2]. It is worth emphasizing that the hormetic stimulatory window of toxic substances and the therapeutic window of pharmaceuticals are conceptually similar [1], and merely reflects different aspects of the same phenomenon. The dominating influence of toxicologists in the debate has probably also contributed to the widespread idea that the low-dose effect in hormesis is generally an adaptive response, an assumption that evidently makes most sense in a toxicological perspective.

Another matter of debate, which also needs to be addressed when using the term “hormesis”, is its universality. The keenest proponents of hormesis have argued that hormesis is actually a more general phenomenon than the classical, well-established threshold theory, and should therefore be considered the default when assessing dose-response relations [2]. Although it seems plausible that hormetic phenomena are more common than hitherto demonstrated, and although advocating the search for hormesis by using wide ranges of concentrations is much deserving, it seems as yet unwarranted to claim that hormesis is universal since the phenomenon probably relies on different mechanisms in different instances and therefore is highly context-dependent. Moreover, the claim for its superiority to the threshold model and its generalizability has probably fuelled much of the recent skepticism towards the concept of hormesis [1,22].

Thus when in the remaining article referring to hormesis, we adhere to the definition suggested by Kendig [1], and view hormesis as a dose-dependent bidirectional effector-endpoint relation, which is unrelated to the mechanism and should not, although seemingly common and underappreciated, be considered universal.

## 3. Female Sex Hormones and Hormesis

**Figure 2:**
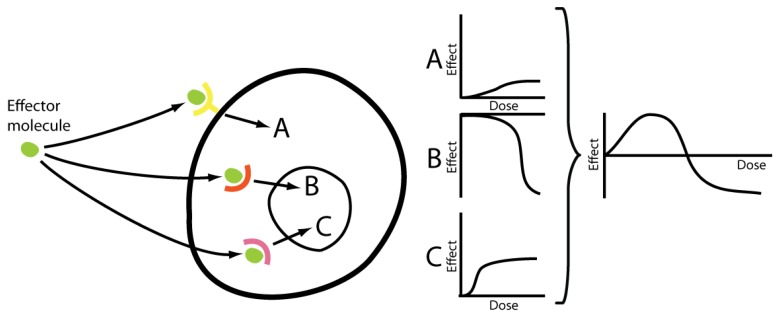
Given the fact that female sex hormones exert their effects through multiple pathways, differing in potency and effective concentration range, it is reasonable that when these are taken together, a more complex, e.g. hormetic, dose-response pattern can occur. **(A)**, **(B)** and **(C)** correspond to different signal pathways in this hypothetical model, providing a theoretical mechanistic framework for hormetic dose-response relations.

Progestagens and estrogens both exert their effects through multiple pathways, each of which may constitute highly complex signal systems. Progestagens mainly act via the two nuclear progesterone receptors A and B, which are both derived from the same gene [31], but often oppose each other's effects [32]. There is also evidence of membrane-bound progesterone receptors [33,34], even though the pharmacological importance of these remains to be proven. In the case of estrogens, the classical pathway – the nuclear estrogen receptors α and β (ERα and ERβ) [35] – are complemented by effects mediated by membrane bound receptors, such as GPR30 [36], and also by direct effects including redox cycling [37]. Further, at very high doses, 17β-estradiol is known to cause down-regulation of its own receptors [38] at the same time as stimulating other receptors of the nuclear receptor superfamily, thus activating a totally different set of genes in the toxicological compared to the physiological concentration range [39]. It has furthermore been speculated that different subsets of membrane receptors, e.g. defined by their residence in membrane caveolae or lipid rafts, can result in non-monotonic dose-response relations [40]. These multifaceted signal systems in turn affect a wide range of biological mechanisms, thus further adding to the intricacy of estrogens' and progestagens' effects. Given this complexity, far from the single-receptor situation which is the basis of the linear dose-response model, it is not unexpected that female sex hormones frequently produce hormetic phenomena. As aforementioned, complex signal pathways is what mechanistically allows hormesis to occur [30] (Figure 2).

There are numerous examples of estrogen and progestagen hormesis affecting a wide variety of endpoints, including cerebral ischemia [16,17], calcium content in bones [41], bone development [42], dopamine transporters and release [43,44], mammary gland differentiation [45,46], capillary endothelial cellular adhesion [47], plasminogen activator regulation [48], DNA synthesis in endothelial cells [49], insulin sensitivity [50], genital development [51-53], growth of cultured tumor cells [54-56], cardiac monophasic action potentials [57], levels of cytosolic magnesium ions [58], anxiety [59,60], sulfotransferase activity in cancer cells [61] and multiple inflammatory processes [62-67]. A few of these are presented below in detail to further highlight the hormetic potential of estrogens and progestagens.

### 3.1. Cerebral Ischemia

In 2005, Theodorsson *et al.* published a study originally designed to investigate if the earlier reported neuroprotective effects of estrogens could be explained by effects on the neuropeptide galanin [12]. Numerous earlier animal stroke experiments had demonstrated neuroprotective effects of estrogens [7,68-70], however in this study 17β-estradiol unexpectedly turned out to be damaging [12]. This raised the question of what methodological factor could be responsible for the diametrical discrepancy in results, and differences in estrogen administration regimes was in an early phase suspected to be the culprit. In the study by Theodorsson and Theodorsson a certain type of subcutaneously implanted slow-release pellets from the company Innovative Research of America (IRA) was used for administration of the hormone. Two subsequent trials investigated this method and the two other commonest methods for estrogen administration, and it was found that the IRA pellets in fact were exceptional in producing highly supraphysiological, long-lasting serum concentration peaks of 17β-estradiol, while the other methods rendered physiological levels or a pattern of short peaks [71,72]. Soon thereafter a meta-analysis of methods in estrogen-stroke rat experiments was published reporting that the high-dose IRA pellets were in fact the only methods capable of inducing increased damage, and that higher dosed pellets were most likely to be detrimental [16]. The hypothesis was later also experimentally validated [17]. Hence, it is very likely that at least part of the controversy of estrogens' effect in animal stroke models was caused by a hormetic phenomenon that remained unnoticed for a long time due to underestimation (including ours) of the importance of well-established administration regimens.

The mechanisms for estrogens' protective effects are probably multifactorial, including decreased apoptosis, decreased inflammation, beneficial vascular effects and growth factor modulation [37,73,74]. This has been given much attention because of the strong potential of the hormone as a neuroprotectant, contrasting the possibly detrimental effects of estrogens in stroke, for which few mechanistic suggestions have been investigated. However, in a recent review, it was hypothesized that hormetic effects of estrogens on inflammation could be the mechanism behind the hormone's paradoxical effects on stroke. This hypothesis was based on an assessment of several rat experimental studies showing increased [13,78,79] or decreased [80-82] cerebral inflammation in the light of earlier studies investigating the produced serum concentrations [71] and effects [16,17] of different estrogen administration regimes [67].

Differential effects of estrogens on stroke have, as mentioned earlier, not only been reported in animal models. An analogous controversy is obvious when assessing results from the larger studies in human populations [3-5,11,75]. Although the doses used in these different studies are somewhat too similar to each other to draw any conclusions about hormetic effects, it must be seen as possible that the U-shaped dose-response curves seen in animal models could also be relevant in human estrogen consumption in general, and HT in particular.

Concerning progesterone, it has likewise been suggested (however on relatively weak grounds) that low doses could be protective and higher doses could increase risk of cerebral ischemia due to the hormone's bidirectional, dose-dependent effects on cytosolic magnesium ions in cerebral vascular smooth muscle cells. Normal concentrations of cytosolic magnesium ions, which are sustained by low levels of female sex hormones, are beneficial for vascular function, while depletion of these ions, caused by high levels of progestagens, result in cerebral vasospasm. The vasospasm leads to decreased cerebral blood perfusion, which could be related to migraine headaches and perhaps also an increased risk of stroke [58].

### 3.2. Inflammation

Many of the known examples of hormesis need pharmacological manipulation of the active substance to appear. However, when it comes to estrogens' effects on inflammation, hormesis-like phenomena can actually be observed *in vivo* during pregnancy. Non-pregnant women are more Th1-tilted than men are, which has been assessed as an estrogenic effect, while the shift from Th1 to the antagonizing Th2 that appears during pregnancy has also largely been attributed to changes in female sexual steroids [76]. Hence it seems that, even under physiological conditions, paradoxical suppression/potentiation of different parts of the immune system results from different concentrations of estrogens, which is compatible with the concept of hormesis, and thus it is easy to imagine that pharmacological hormone manipulations even more potently can exert such phenomena. Numerous studies have been dedicated to experimentally investigate estrogens' effects on inflammation, and the results reveal an almost unbelievable complexity, that however to a large part can be understood as consequences of the fairly logical overall effects of estrogens in pregnancy, aimed at avoiding abortion of the fetus [66].

Most experimental studies demonstrating hormetic phenomena of female sex hormones on inflammation suggest that low hormone concentrations are pro-inflammatory whereas high hormone concentrations are anti-inflammatory [63], such as the effects of estrogens on the pro-inflammatory cytokine IL-1 [77]. Similar results have been reported when it comes to the effects on TNF [62,64,65], natural killer cells and adhesion molecules, all seeming to be inhibited by high estrogen and/or progestagen levels while being stimulated by low levels [66]. Furthermore, inhibition of immune cell apoptosis has been demonstrated in lower levels than have the opposite [66]. These observations seem to be well in line with the understanding of the anti-inflammatory role of the high estrogen concentrations during pregnancy. However, the complexity increases even more when the effects of estrogens on a broader range of cytokines is taken into consideration, since not only concentration, but also the type of effector cell, the cytokine milieu and other factors seem to be crucial [66]. As abovementioned it was in a recent review hypothesized that high dose estrogen administration regimens could increase cerebral inflammation while low dose regimens exert the opposite effect, which is quite contrary to the pattern presented above. The discordant patterns concerning in which concentration-ranges estrogens are neuroprotective or neurotoxic could possibly result from organ differences or reflect discrepancies in the measured end-points.

In this section, the aspect of estrogen type also merits attention, even though it does not present a clear example of hormesis. Interestingly, CEE, which was used in WHI and then resulted in increased risk of stroke [11,75], has in several studies been reported to be pro-inflammatory in contrary to 17β-estradiol [66], which has been administered in virtually all animal studies reporting decreased risk of stroke [16]. CEE regimes are generally considered more potent than 17β-estradiol regimes, even if the exact potency is difficult to compare.

Even though estrogens' actions on inflammation in different organ and cells are exceedingly complex and it is difficult to draw any firm conclusions, it is clear that estrogens are highly capable of exerting hormetic effects also in inflammation, and that this needs to be taken into account when studying relevant phenomena. Hormetic effects on inflammation are particularly interesting since they are not only relevant for, as aforementioned, stroke and obviously inflammatory disorders such as rheumatoid arthritis, but also for e.g. cardiovascular diseases, osteoporosis and cancer.

### 3.3. Cardiovascular Disease

Hormetic effects of female sex hormones on inflammation have, as aforementioned, far-reaching implications for cardiovascular diseases, since inflammation e.g. is a central process in the pathogenesis of atherosclerotic plaques [83] and in the development of myocardial infarction [84]. However, there are also more specific examples of hormesis that can be relevant, such as the effects on the anticoagulant protein plasminogen activator. In a cell culture experiment using bovine aortic endothelial cells the effects of 17β-estradiol and progesterone on plasminogen activator was investigated. It was found that even though 17β-estradiol and progesterone concentrations corresponding to low physiological *in vivo* levels activated the protein, higher concentrations inhibited it, and thus it was concluded that in this respect, estrogens and progesterone in pharmacological doses can be thrombogenic [48].

Further, estrogens' effects on the DNA production in endothelial cells have been reported to obey hormetic principles. In a human umbilical smooth muscle cell line it was found that 17β-estradiol in physiological concentrations stimulated [^3^H]thymidine incorporation into DNA whereas pharmacological concentrations were inhibitory. These findings may have bearing on cardiovascular diseases because of the role of smooth muscle cells in atherosclerosis pathophysiology [49].

Insulin resistance is a prominent feature of the metabolic syndrome and thus intimately related to cardiovascular diseases. In a RCT of the effects on conjugated equine estrogens on insulin sensitivity in postmenopausal women it was shown that the standard dose of 0.625 mg/day increased while 1.25 mg/day decreased insulin sensitivity [50]. This is a clear demonstration that the hormetic effects of female sex hormones can indeed prevail in clinical situations.

Another relevant example concerns arrhythmia, which is a central feature of several cardiac diseases. It has been reported that progesterone's modulation of action potentials in heart muscle also displays hormetic patterns, so that while low progesterone levels were found to shorten action potentials, high doses lengthened the same [57].

### 3.4. Anxiety

Progestagens have in several studies been reported to exert both anxiogenic and anxiolytic effects, in accordance with hormetic principles. Both animals and postmenopausal women treated with the progestagen allopregnanolone exhibit anxiogenic responses in the lower and anxiolytic responses in the higher dose-spectrum [59]. These effects are supposedly mediated via allopregnanolone's effects the GABA(A) receptor [59]. In a study of mouse behavior in a plus-maze, it was shown that low doses of pregnanolone sulfate increased anxiety, while high doses on the contrary were anxiolytic [60], which matches pregnanolone sulfate's bidirectional effects on the GABA(A) receptor [85].

## 4. Conclusions

Hormesis is a highly relevant concept for female sexual steroids' effects on many biological endpoints. This is particularly evident concerning estrogens, but there are also several examples of progestagen hormesis. The dominance of estrogens over progestagens concerning number of reported cases of hormesis probably reflects that the former hormone has been subjected to more intense research efforts, but can also be due to an actually stronger tendency of estrogens to produce hormetic dose-response relations.

Therefore experiments designed to elucidate the proper biological and therapeutic effects of female sex hormones should be performed with hormesis in mind. A wide range of doses should be adopted, and importantly, the biological relevance of these doses must be assessed by serum hormone measurements and subsequent comparison with the intended clinical/biological situation. Since HT is mainly given in a low dose range, it seems reasonable to primarily aim at simulating these levels, and it is of utmost importance that the measurements of hormone after hormone manipulation, e.g. in an animal, is performed in several time-points [17]. The same caution of using several dose levels – though difficult – may also be of relevance in human studies where a single dose has hereto been the rule.

## Figures and Tables

**Table 1:** Literature review of 100 studies where 17β-estradiol has been administered to rats or mice.

**Number of 17β-estradiol doses**	**Number of studies**	**Number of occasions of 17β-estradiol measurement**	**Number of studies**
1	86	0	73
2	7	1	25
3	3	2	0
4	2	3	0
5	1	4	2
6	0		
7	1		
